# Mechanical jejunal obstruction caused by a migrated intragastric balloon: a case report

**DOI:** 10.1093/jscr/rjae744

**Published:** 2024-11-24

**Authors:** Mohamed S Ghali, Osman O Elhassan, Raed M Al-Zoubi

**Affiliations:** Department of Surgery, Acute Care Surgery, Hamad Medical Corporation,3050, Doha, Qatar; Department of General Surgery, Ain Shams University, 38 Abbassia, 1181, Cairo, Egypt; Department of Medical Education, Hamad Medical Corporation (HMC), 3050, Doha, Qatar; Surgical Research Section, Department of Surgery, Hamad Medical Corporation, 3050, Doha, Qatar; Department of Biomedical Sciences, QU-Health, College of Health Sciences, Qatar University, 2713, Doha, Qatar; Department of Chemistry, Jordan University of Science and Technology, 22110, Irbid, Jordan

**Keywords:** intragastric balloon, small bowel obstruction, laparoscopy

## Abstract

An intragastric balloon is often used as a temporary solution for weight loss. It is endoscopically placed into the stomach and filled with air or saline to encourage fullness and reduce meal intake. A 23-year-old female with a history of a gastric balloon procedure presented to the emergency department with generalized abdominal pain and recurrent vomiting. Initial imaging with ultrasound and computed tomography scans revealed a collapsed migrated gastric balloon causing a small bowel obstruction. Despite initial conservative management, the patient required surgical intervention, which involved laparoscopic exploration, mini laparotomy, and enterotomy to extract the migrated balloon. Postoperatively, the patient had an uneventful recovery and was discharged with a stable condition. This case underscores the importance of considering device-related complications in patients with gastric balloons presenting with gastrointestinal symptoms and highlights the need for prompt imaging and appropriate surgical management.

Intragastric balloon migration leading to small bowel obstruction is a rare but serious complication that should be considered in patients presenting with abdominal pain and vomiting following a gastric balloon procedure. Prompt imaging and surgical intervention are crucial for effective management and favorable outcomes.

## Introduction

Placing an intragastric balloon (IGB) is a common non-surgical weight loss technique used to treat obesity [1]. Prior surgery, IGBs are frequently utilized as a temporary solution for bridging therapy. In order to encourage a feeling of fullness and decrease food intake, a silicone balloon is inserted endoscopically into the stomach and filled with air or saline. Although they are usually regarded as harmless, complications such as balloon rupture, migration, and consequent gastrointestinal blockage might occur [2].

This case report details the presentation, diagnosis, management, and outcome of a young female who experienced a small bowel obstruction due to the migration of a previously placed gastric balloon.

## Case history

A 23-year-old female had a history of a gastric balloon procedure performed one year ago in a private hospital, but there was no follow-up. She presented to the emergency department with complaints of generalized abdominal pain, recurrent vomiting, and constipation, but she was able to pass flatus. The abdominal pain was non-radiating, and the vomiting had rendered her unable to eat. She did not report loose motions, fever, or urinary symptoms. On physical examination, she had abdominal distension, a soft, lax abdomen with mild tenderness, and mildly exaggerated bowel sounds, but otherwise she was vitally stable.

## Initial evaluation

**Figure 1:**
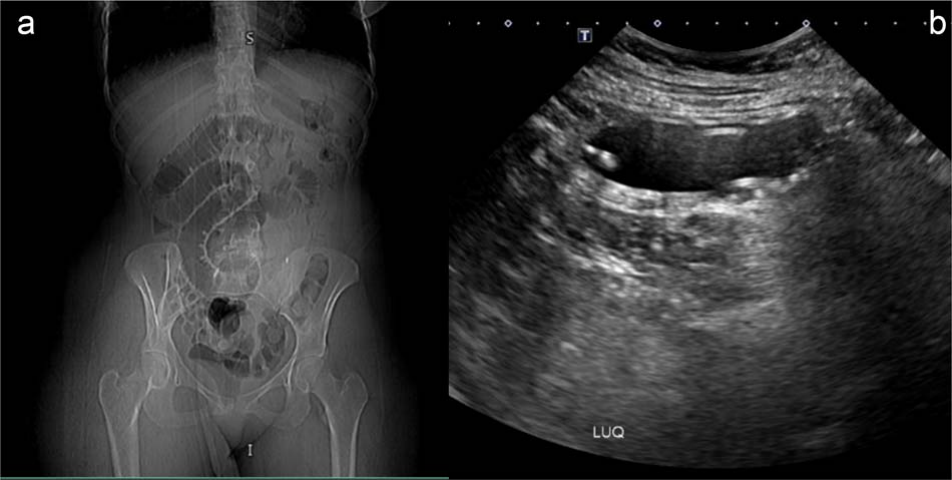
(a) Plan X-ray abdomen showing dilated jejunal loops. (b) Abdominal US showing small bowel containing IGB.

After she arrived in the emergency room, plan X-ray abdomen showed dilated jejunal loops and an ultrasound (US) of her abdomen was carried out, and it was discovered that the gastric balloon in the left upper quadrant of the stomach lumen had drastically shrunk, indicating that it may have ruptured (Fig. 1). Nevertheless, the balloon was not visible during the upper endoscopy that she had later.

**Figure 2:**
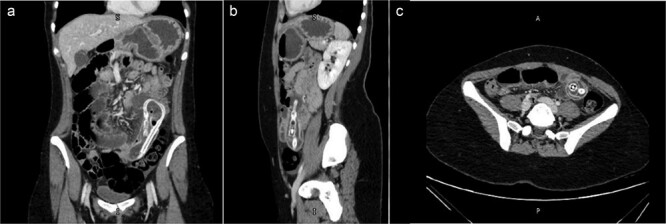
(a) Coronal view (b) sagittal view (c) axial view of the abdominal CT scan with oral water-soluble contrast showing IGB migrated and impacted in the jejunum with the proximal SBO.

Following the initial evaluations, the patient had an urgent computed tomography (CT) abdominal scan, which revealed that the migrating collapsed gastric balloon, which was trapped in the small bowel loops with proximally dilated loops, was the cause of the small intestinal obstruction (SBO) (Fig. 2). The migrating IGB was determined to be the source of the patient's small intestinal blockage. As the patient was hemodynamically stable and the goal was for the balloon to transit naturally down the alimentary tract, the surgical team advised conservative care at this point.

## Surgical intervention

**Figure 3:**
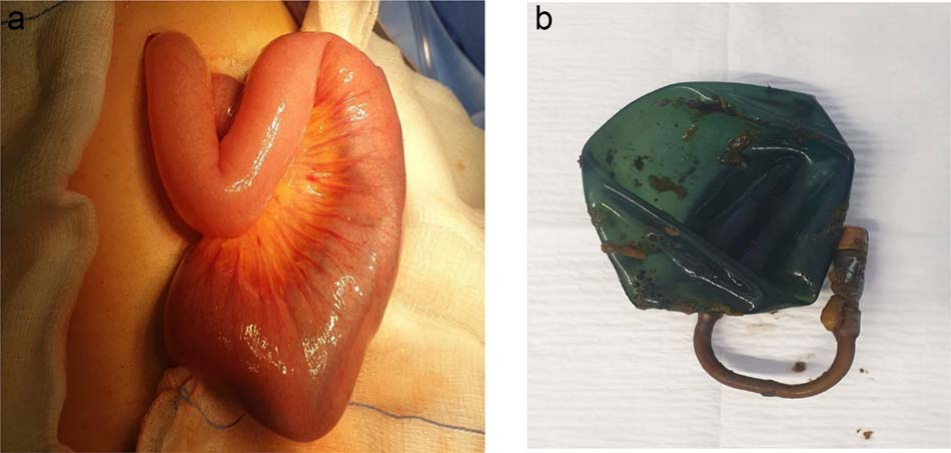
(a) Small bowel loop containing IGB. (b) IGB extracted from the small bowl.

Despite conservative efforts, the patient did not show any improvement after five days from admission, so surgical intervention became necessary. The patient underwent laparoscopic exploration, where we identified the area of small bowel obstruction (SBO), and we appreciated the balloon in it. A mini laparotomy through widening of the umbilical port to avoid soiling after the enterotomy to extract the migrated intragastric balloon. The balloon was found in the jejunal loop, causing dilatation of the small bowel proximal to the obstruction and collapse distally. There was free fluid in the pelvis and hyperemia was observed at the balloon location. Following proximally milking the gastric balloon away from the obstruction and hyperemic bowel, a longitudinal incision was made for the enterotomy, the balloon was removed, and the enterotomy site was closed transversely to prevent bowel stricture (Fig. 3). The repaired bowel was returned back to the abdomen and abdominal wound was closed in layers. Since the field was pristine and there had not been significant bowel content soiling, we chose not to install a drain in this case.

On the fifth postoperative day, the patient's condition stabilized, and she was allowed to go home with regular bowel movements and good diet tolerance. After a follow-up evaluation at the clinic after a fortnight, the patient was allowed to return to her usual activities.

## Discussion

Obesity, a major global health issue, increases the risk of various serious diseases. To address this, several treatments, both invasive and non-invasive, have been developed [3], including IGBs, bariatric surgery, and lifestyle changes. Since their introduction in the 1980s [4], IGBs have been used to promote weight loss by reducing oral intake and enhancing satiety in a non-surgical manner [3]. Their appeal lies in the ease of insertion, adjustment, and removal. However, IGBs can lead to complications, with one of the most serious being migration causing intestinal obstruction. Although rare, balloon migration can lead to SBO, requiring urgent surgical intervention [5].

Minimally invasive techniques like endoscopy are preferred for obese patients due to the reduced risk of complications compared to open surgery. These complications can include infections, wound issues, and extended hospital stays [6]. In this case, a saline-filled IGB spontaneously deflated, migrated from the stomach to the small bowel, and caused a complete SBO. While open surgery is commonly reported for such cases, this patient underwent successful laparoscopic removal, highlighting the benefits of minimally invasive procedures.

Primary laparoscopic repair is favored if the bowel tissue is not damaged, but resection is needed if gangrene is present. Although technically demanding, laparoscopic techniques are advantageous for obese patients, resulting in fewer complications and shorter recovery times [6–8].

IGBs can obstruct the bowel at various levels, from the duodenum to the rectum. Previous cases have reported blockages at the sigmoid colon and terminal ileum, but this case involved the jejunal loop. Early diagnosis, prompt surgical intervention, and patient awareness of IGB risks are crucial for managing such complications. Regular follow-up and removal within six months are recommended to prevent these issues [9].

## Conclusion

This case highlights and confirms the complications that can arise from a gastric balloon procedure, a non-surgical treatment commonly used for weight loss. If the patient is presented with symptoms indicative of an obstructive process after IGB insertion, IGB migration-causing obstruction should be on top of the differential. Patient counseling before the IGB procedure is of high importance, with a focus on its potential complications and the importance of follow-up for timely removal. This case serves as a critical reminder for clinicians to maintain a high index of suspicion for IGBs-related complications in similar clinical scenarios.

## Data Availability

Data will be made available on request.
